# Supplementary material for: Design and Validation of an Instrument for Evaluating Training and Education for Health and Proper Use of Vaccines “VACUNASEDUCA”
Source: Int J Environ Res Public Health. 2021 Jul 8;18(14):7321. doi: 10.3390/ijerph18147321 (PMC8304289; doi:10.3390/ijerph18147321)
Supplement: Supplementary file 1 [file ijerph-18-07321-s001.zip › ijerph-1207152-supplementary.pdf]

EDAD	GÉNERO	PAÍS	PROFESIÓN	P01	P02	P03	P04
2	1	Albania	1	3	3	3	2
2	0	Albania	1	2	3	3	2
3	1	Albania	1	3	3	1	3
2	1	Alemania	1	3	3	1	1
1	0	Alemania	1	1	3	1	3
2	0	Alemania	3	3	3	3	3
3	0	Alemania	3	3	3	3	3
4	0	Alemania	3	3	3	3	3
3	1	Alemania	1	3	3	3	3
3	1	Alemania	1	3	3	3	3
3	0	Alemania	3	1	3	3	3
2	1	Alemania	1	3	3	3	3
3	0	Alemania	1	3	3	3	2
3	1	Alemania	3	3	3	3	2
2	1	Alemania	1	2	3	3	3
3	1	Alemania	1	3	2	2	2
2	0	Alemania	1	2	3	3	3
2	0	Alemania	1	3	3	1	3
2	1	Alemania	1	2	2	3	3
3	0	Alemania	3	3	3	3	3
3	1	Alemania	3	3	3	3	3
4	0	Alemania	1	2	3	3	3
4	1	Alemania	1	2	3	3	3
2	1	Alemania	1	3	3	3	2
3	1	Alemania	1	2	3	3	3
3	1	Alemania	1	2	2	3	3
2	1	Alemania	1	3	2	3	3
3	1	Alemania	1	3	3	3	3
1	1	Alemania	1	3	3	3	3
1	0	Alemania	1	3	3	3	3
1	0	Alemania	2	3	3	3	3
2	0	Andorra	3	3	3	3	3
3	1	Andorra	3	3	3	3	3
2	1	Angola	1	3	3	2	3
4	1	Angola	1	3	3	1	1
3	0	Angola	1	3	1	1	2
2	1	Angola	1	2	2	3	3
3	0	Angola	1	2	3	3	3
4	0	Arabia Saudita	1	3	3	3	2
3	0	Arabia Saudita	1	3	3	3	3
3	0	Arabia Saudita	3	3	3	3	3
2	0	Arabia Saudita	3	3	3	3	3
4	1	Argelia	1	3	2	2	2
1	0	Argelia	1	1	3	1	1
2	0	Argentina	1	3	3	3	3
2	0	Argentina	2	3	3	3	3
2	1	Argentina	1	3	3	3	3
2	0	Argentina	1	2	1	2	2
3	0	Argentina	1	2	2	2	3

3	0	Argentina	2	3	3	3	3
3	1	Australia	1	3	3	2	3
3	1	Australia	2	3	3	3	3
3	1	Austria	2	3	3	3	3
4	0	Austria	1	3	3	3	2
3	1	Austria	2	3	3	3	3
1	1	Austria	1	3	3	3	3
2	1	Austria	3	2	3	3	3
2	1	Austria	3	2	2	3	3
2	1	Bangladés	1	3	3	1	3
1	1	Bangladés	1	3	3	1	3
3	0	Bélgica	1	1	3	1	2
1	0	Bélgica	1	3	3	2	3
2	0	Bélgica	2	3	3	3	3
3	1	Bélgica	1	3	3	3	2
3	0	Bélgica	3	3	3	3	3
2	1	Bélgica	3	2	3	2	2
3	1	Bolivia	2	3	2	2	2
2	0	Bolivia	2	3	3	3	2
1	0	Bolivia	1	1	3	1	3
1	0	Bolivia	1	1	3	1	1
1	0	Bolivia	3	2	2	2	3
2	0	Bosnia-Herzegovina	1	3	3	3	3
2	1	Botsuana	1	3	3	2	2
3	0	Brasil	2	3	3	3	2
3	1	Brasil	1	3	3	3	1
3	1	Brasil	1	3	3	3	3
1	1	Brasil	1	3	3	3	3
2	1	Brasil	1	2	1	1	3
2	1	Brasil	1	2	2	2	2
2	1	Brasil	2	2	2	2	3
2	0	Brasil	2	2	2	2	3
3	1	Bulgaria	1	3	3	3	3
3	0	Bulgaria	1	3	3	3	3
3	1	Bulgaria	1	3	3	3	3
3	1	Bulgaria	1	1	3	3	3
2	1	Bulgaria	1	1	3	3	3
2	0	Bulgaria	3	2	2	2	2
2	0	Cabo Verde	1	3	3	3	3
2	0	Camerún	1	3	3	3	2
2	1	Camerún	1	3	3	3	2
2	1	Camerún	1	3	3	2	2
2	1	Camerún	1	2	1	1	3
3	1	Canadá	1	3	3	3	3
3	0	Canadá	1	3	3	3	2
3	0	Canadá	1	3	3	3	3
3	0	Canadá	1	3	3	3	3
2	0	Canadá	1	2	3	3	3
3	1	Canadá	2	3	3	3	2
4	0	Chile	1	3	3	3	3

3	1	Chile	1	3	3	3	3
3	0	Chile	1	3	3	3	3
2	1	Chile	1	3	3	3	3
2	1	China	3	2	2	2	3
4	0	China	3	3	3	1	1
4	0	China	3	3	3	3	3
3	0	China	1	1	3	3	1
3	0	China	1	1	3	3	3
3	0	China	1	1	3	3	3
3	0	Chipre	1	2	3	3	3
3	0	Chipre	1	3	3	2	3
3	0	Chipre	1	3	3	3	3
2	1	Chipre	1	3	2	2	3
4	0	Colombia	1	3	3	2	2
3	0	Colombia	1	3	3	3	3
2	1	Colombia	3	3	3	3	3
3	1	Colombia	1	3	3	3	3
3	0	Colombia	1	3	3	3	3
3	1	Colombia	1	3	3	2	3
2	0	Colombia	1	2	2	2	2
2	1	Colombia	1	2	2	3	3
4	0	Colombia	1	2	3	3	3
3	1	Colombia	2	3	3	3	3
3	1	Colombia	2	2	3	2	3
3	1	Colombia	2	3	2	3	3
3	0	Colombia	2	2	2	2	2
2	0	Costa de Marfil	1	2	2	2	2
2	0	Costa de Marfil	3	2	3	2	2
1	0	Cuba	1	3	3	3	2
2	0	Cuba	1	2	3	3	3
3	0	Cuba	3	2	2	2	2
2	1	Dinamarca	3	3	3	3	1
2	0	Dinamarca	3	3	3	3	3
3	0	Dinamarca	1	3	3	3	3
2	0	Dinamarca	1	3	3	3	3
1	0	Dinamarca	1	3	1	3	3
2	1	Dinamarca	1	3	3	3	1
2	0	Dinamarca	1	3	3	3	1
2	1	Dinamarca	1	3	3	1	1
3	1	Ecuador	1	3	3	3	3
3	1	Ecuador	1	3	3	3	3
2	0	Ecuador	1	2	3	3	3
2	0	Ecuador	1	2	3	3	3
3	0	Ecuador	1	2	2	2	2
3	0	Ecuador	1	2	2	2	3
3	1	Emiratos Árabes Unidos	1	3	3	3	3
3	0	España	1	3	3	3	3
1	1	España	1	2	3	3	3
3	0	España	1	3	3	3	3
2	1	España	1	3	3	3	3

3	1	España	1	3	3	1	2
3	1	España	1	3	3	1	1
3	1	España	1	3	2	2	2
3	1	España	1	2	2	2	3
3	1	España	1	3	2	3	3
3	1	España	1	2	3	3	3
3	1	España	3	2	3	3	3
4	1	España	3	3	3	3	3
3	1	España	1	3	3	3	3
2	1	España	1	3	2	3	3
2	0	España	1	2	2	2	2
1	0	España	3	2	2	2	2
1	0	España	1	2	3	3	3
2	1	España	1	3	3	3	3
2	1	España	1	3	3	3	2
3	1	España	1	3	3	3	3
3	0	España	3	3	2	3	3
3	0	España	2	3	2	3	3
1	1	España	1	3	3	3	3
2	0	España	1	2	2	3	3
3	0	España	3	3	3	3	3
3	0	España	3	3	1	3	3
2	1	España	3	3	3	3	3
3	1	España	3	3	3	3	3
3	0	España	3	2	3	3	3
2	0	España	3	3	3	3	3
2	1	España	2	2	3	2	3
2	1	España	2	3	3	3	3
2	1	España	1	3	3	3	3
2	1	España	1	3	3	3	3
2	1	España	1	3	3	3	3
2	0	España	1	3	3	3	3
2	0	España	1	3	3	3	3
2	0	España	1	3	2	2	3
2	0	España	1	3	3	3	3
2	0	España	1	3	2	2	3
2	0	España	1	3	3	3	3
2	1	España	1	3	3	3	3
2	1	España	1	3	3	3	3
2	1	España	1	3	3	2	2
2	1	España	1	3	3	3	2
2	1	España	1	3	3	3	3
2	1	España	1	3	3	3	3
1	0	España	1	3	2	2	2
2	0	España	1	3	3	3	3
2	0	España	1	3	2	2	2
2	0	España	1	3	3	3	3
2	0	España	1	3	3	3	3
2	0	España	1	3	2	2	2
2	0	España	1	2	2	2	2

2	0	España	1	3	3	3	3
2	0	España	1	2	2	2	2
2	0	España	1	3	3	2	3
2	0	España	1	3	3	3	3
2	0	España	1	2	2	2	2
2	0	España	1	3	3	3	3
2	0	España	1	3	3	3	3
2	0	España	1	2	3	3	3
2	0	España	1	3	3	2	2
2	0	España	1	3	3	2	3
2	0	España	1	3	3	3	3
2	0	España	1	2	2	3	2
2	0	España	1	2	2	2	2
2	0	España	1	2	2	3	3
2	0	España	1	3	3	2	2
2	0	España	1	3	2	3	3
2	1	España	1	3	3	3	3
3	1	España	1	2	2	2	3
2	1	España	1	3	3	3	3
2	0	España	3	3	3	3	2
3	0	España	3	3	3	3	2
2	0	España	1	3	3	3	3
3	0	España	1	3	3	3	3
2	0	España	1	3	3	3	2
2	0	España	1	2	3	3	3
2	0	España	1	3	3	3	3
3	0	España	1	2	3	3	3
2	1	España	1	3	3	3	3
3	1	España	1	2	2	3	3
3	1	España	1	3	3	3	3
3	1	España	1	3	3	3	2
3	1	España	1	2	2	3	2
3	1	España	1	3	3	3	3
2	1	España	1	3	3	3	3
2	1	España	1	3	3	3	3
2	0	España	1	3	3	3	3
2	0	España	1	3	3	3	3
2	0	España	1	3	3	3	3
2	0	España	1	2	2	3	3
2	0	España	1	2	2	2	3
2	0	España	1	3	3	3	3
2	0	España	1	3	3	3	3
2	0	España	1	3	3	3	3
2	0	España	1	3	3	3	3
2	1	España	1	3	3	3	3
3	0	España	1	2	2	2	3
3	1	España	1	3	3	2	3
3	0	España	1	3	3	3	3
3	1	España	1	2	3	3	3
3	0	España	1	2	2	2	3

3	0	España	1	3	3	3	3
3	0	España	1	3	3	2	3
3	0	España	1	3	3	3	3
3	0	España	1	3	3	3	3
3	1	España	1	2	2	3	3
3	1	España	1	3	3	3	3
3	1	España	1	3	3	3	3
3	1	España	1	3	3	2	3
3	1	España	1	3	3	3	2
3	0	España	1	3	3	3	3
3	0	España	1	3	3	3	2
3	0	España	1	3	3	3	2
3	0	España	1	3	3	3	3
3	0	España	1	3	3	2	2
3	0	España	1	3	3	3	2
3	0	España	1	3	3	3	3
3	0	España	1	3	3	3	3
3	0	España	3	3	3	3	3
3	0	España	3	3	3	3	3
3	0	España	3	3	3	3	3
3	0	España	3	3	3	3	3
3	0	España	3	2	2	2	2
3	0	España	1	3	3	3	2
2	1	España	1	3	3	3	3
2	1	España	1	3	3	2	2
2	0	España	1	2	2	2	2
2	0	España	1	3	3	3	3
2	0	España	1	3	2	3	2
2	0	España	2	3	3	3	3
2	0	España	2	2	3	3	3
2	0	España	2	3	3	3	3
2	0	España	1	3	3	3	3
2	1	España	1	3	3	3	3
2	1	España	1	2	3	3	3
2	0	España	1	3	3	2	3
2	1	España	1	2	3	3	3
2	0	España	1	3	3	3	3
2	0	España	1	3	3	3	3
2	0	España	1	3	3	3	3
2	0	España	1	3	3	3	3
2	0	España	1	3	3	3	3
2	0	España	1	3	3	3	3
2	0	España	1	2	2	3	3
2	0	España	1	3	3	3	3
2	0	España	1	3	3	3	3
2	0	España	1	3	3	3	3
2	0	España	1	3	3	3	3
2	0	España	1	3	3	3	2
2	0	España	1	3	3	3	3
2	1	España	1	3	3	3	3

2	1	España	1	3	3	3	3
2	1	España	1	3	3	3	3
3	1	España	1	3	3	3	3
3	1	España	1	3	2	2	3
2	1	España	1	3	3	2	3
2	1	España	1	2	3	3	3
3	1	España	1	3	2	3	2
3	1	España	1	3	3	3	3
3	0	España	1	3	3	3	3
3	0	España	1	3	3	3	3
3	0	España	1	3	3	3	3
3	0	España	1	3	3	3	3
3	0	España	1	3	3	2	3
3	0	España	1	3	3	3	3
3	0	España	1	3	3	3	3
3	0	España	1	3	3	2	3
3	0	España	1	3	3	2	3
3	0	España	3	3	3	3	3
3	0	España	3	2	2	3	3
3	1	España	3	3	3	3	2
3	1	España	3	2	3	3	3
3	1	España	3	3	3	3	3
3	1	España	3	3	3	3	3
3	1	España	3	3	3	3	3
3	1	España	3	3	3	2	3
3	1	España	3	3	3	3	3
3	0	España	3	3	3	3	3
3	0	España	3	3	3	3	2
3	0	España	3	3	3	3	3
3	0	España	3	3	3	3	3
3	0	España	3	3	3	3	3
3	0	España	1	2	2	3	3
3	1	España	1	3	3	3	3
3	1	España	1	3	3	2	3
3	1	España	1	3	3	3	3
3	0	España	1	3	3	3	3
3	0	España	1	3	3	3	3
3	0	España	1	3	3	3	2
3	0	España	1	3	3	2	3
3	0	España	1	3	3	3	3
3	0	España	1	3	3	3	2
3	0	España	1	3	3	2	3
3	0	España	1	3	3	3	3
3	0	España	1	3	3	2	3
3	0	España	1	3	3	3	3
2	1	España	1	3	3	3	3
2	1	España	1	3	3	3	3
2	1	España	1	3	3	3	3
2	1	España	1	3	3	3	3
2	1	España	1	3	3	3	3

2	1	España	1	3	3	3	3
2	0	España	1	3	3	3	2
2	0	España	1	3	3	3	3
2	0	España	1	3	3	3	2
2	0	España	1	3	3	3	3
2	0	España	1	3	3	3	3
2	0	España	3	3	3	3	3
2	1	España	3	3	3	3	3
2	0	España	3	2	3	3	3
2	1	España	3	3	3	3	2
2	0	España	3	3	3	3	3
2	0	España	3	3	3	3	2
2	0	España	3	3	3	3	3
2	0	España	3	3	3	3	3
2	1	España	3	3	3	3	3
3	1	España	3	3	3	3	3
3	1	España	3	3	3	3	3
3	0	España	3	3	3	3	3
3	0	España	3	2	2	3	3
3	0	España	3	3	3	3	3
3	1	España	3	3	3	3	3
3	1	España	1	3	3	3	3
3	1	España	1	3	3	3	2
4	1	España	1	3	2	3	3
2	0	España	2	3	3	3	2
2	0	España	2	3	3	3	2
2	0	España	2	2	3	3	3
1	1	España	2	3	3	3	3
1	0	España	2	2	3	3	3
1	0	España	1	3	3	3	3
1	1	España	1	3	2	3	3
1	1	España	1	3	3	2	3
1	1	España	2	3	3	3	2
1	1	España	1	3	3	3	3
1	1	España	2	3	3	3	3
1	0	España	2	3	3	3	3
1	0	España	2	3	3	3	2
1	1	España	2	3	3	3	3
2	0	España	2	3	3	3	3
1	0	España	2	2	2	3	3
1	0	España	2	3	3	3	3
1	1	España	2	3	3	3	3
1	1	España	2	2	2	2	3
1	0	España	2	3	3	3	3
1	0	España	2	3	3	3	2
1	0	España	2	3	3	3	3
1	1	España	2	2	3	3	3
1	1	España	2	3	3	3	3
1	0	España	2	3	3	3	3
1	1	España	2	2	2	2	3

1	0	España	2	2	3	3	3
1	1	España	2	3	3	3	3
1	1	España	2	2	2	3	3
1	0	España	2	3	3	3	3
1	0	España	2	3	2	3	3
1	0	España	2	3	3	3	2
1	1	España	2	3	3	3	3
1	1	España	2	3	3	2	3
1	0	España	2	3	3	3	3
1	0	España	1	3	3	3	3
1	1	España	1	3	3	3	3
1	0	España	1	3	3	3	3
1	0	España	2	3	3	3	3
1	0	España	2	3	3	3	3
1	0	España	2	1	3	3	3
1	0	España	2	2	2	3	3
1	0	España	2	3	3	2	3
1	1	España	2	3	2	3	3
1	0	España	2	3	3	3	3
1	0	España	2	3	3	3	3
1	0	España	2	3	3	3	3
1	0	España	2	3	3	3	2
1	0	España	2	3	3	3	3
1	1	España	2	3	3	3	3
1	1	España	2	3	3	3	3
1	1	España	2	3	3	3	3
1	0	España	2	3	3	3	3
1	0	España	2	3	3	3	3
1	0	España	1	3	3	3	3
1	1	España	1	2	2	3	3
1	0	España	1	3	3	3	3
2	0	España	1	3	3	2	2
2	0	España	1	3	3	3	3
2	0	España	1	3	3	3	3
2	0	España	1	2	3	3	3
2	0	España	1	3	3	3	3
2	0	España	1	2	2	3	3
2	0	España	1	3	3	3	3
2	0	España	1	3	3	3	3
1	0	España	1	3	3	3	3
1	0	España	1	3	3	3	3
1	0	España	1	3	3	3	3
1	0	España	1	3	3	3	3
1	0	España	1	3	3	3	3
2	0	España	1	3	3	3	3
1	0	España	1	3	3	3	3
1	0	España	1	2	2	3	3
1	0	España	1	3	3	3	3
2	0	España	1	2	2	3	3
2	0	España	1	3	3	3	3

1	0	España	2	3	3	3	3
1	0	España	2	3	3	3	3
1	0	España	2	1	3	3	2
1	0	España	2	3	3	3	3
1	0	España	2	3	3	3	3
1	0	España	2	3	3	3	3
1	0	España	2	3	3	3	3
1	0	España	2	3	3	3	3
1	0	España	2	3	3	3	2
1	0	España	2	3	3	3	3
1	0	España	2	2	3	3	3
1	1	España	2	3	3	3	3
2	1	España	2	3	3	3	3
2	0	España	2	3	3	3	3
1	0	España	2	3	3	3	2
1	0	España	2	3	3	3	3
1	0	España	2	3	3	3	2
1	1	España	2	2	2	3	3
1	0	España	2	3	3	3	3
1	0	España	2	3	3	3	3
1	0	España	2	3	3	3	3
1	0	España	2	3	3	3	3
1	0	España	2	3	3	3	3
1	0	España	2	2	2	3	3
1	0	España	2	3	3	3	3
1	0	España	2	3	3	3	3
1	0	España	2	3	3	3	3
1	0	España	2	2	3	3	3
1	0	España	2	3	3	3	3
2	0	España	2	3	3	3	3
2	0	España	2	3	3	3	3
2	0	España	2	3	3	3	3
2	0	España	2	3	3	3	3
2	0	España	2	3	3	3	3
1	0	España	2	3	3	3	3
3	0	España	2	3	3	3	3
3	0	España	2	3	3	3	3
1	0	España	2	2	3	3	3
1	1	España	2	3	3	3	3
1	0	España	2	3	3	3	3
1	0	España	2	3	3	3	3
1	0	España	2	3	3	3	3
1	0	España	2	3	3	3	3
1	0	España	2	3	3	3	3
1	0	España	2	3	3	3	3
1	0	España	2	3	3	3	3
1	0	España	2	3	3	3	3
1	0	España	2	2	2	3	3
1	0	España	2	3	3	3	3
1	0	España	2	3	3	3	3

1	0	España	2	3	3	2	3
1	0	España	2	3	3	3	3
1	0	España	2	3	3	3	3
1	0	España	2	3	3	3	3
1	0	España	2	3	3	3	3
1	0	España	2	3	3	3	3
1	0	España	2	3	3	3	3
1	0	España	2	3	3	3	3
1	0	España	2	3	3	3	3
1	0	España	2	3	3	3	3
1	0	España	2	3	3	3	3
1	0	España	2	3	3	3	2
1	0	España	2	3	3	3	3
2	0	España	2	3	3	3	3
2	1	España	2	3	3	3	3
1	1	España	2	3	3	3	3
1	1	España	2	3	3	3	3
1	1	España	2	3	2	3	3
1	0	España	2	3	3	3	3
1	0	España	2	3	3	3	3
1	0	España	2	2	3	3	3
1	0	España	2	3	3	3	3
1	0	España	2	3	3	3	3
1	0	España	2	3	3	3	3
1	0	España	2	3	3	3	3
1	0	España	2	3	3	3	3
1	0	España	2	3	3	3	3
1	0	España	2	3	3	3	3
1	0	España	2	3	3	3	3
1	0	España	2	3	3	3	3
1	0	España	2	3	3	3	3
2	0	España	2	3	3	3	3
2	0	España	2	3	3	3	3
1	0	España	2	3	3	3	3
1	0	España	2	3	3	3	3
1	0	España	2	3	3	3	3
1	0	España	2	3	3	3	3
1	0	España	2	3	3	3	3
1	0	España	2	3	3	3	3
1	0	España	2	3	3	3	3
1	0	España	2	3	3	3	3
1	0	España	2	3	3	3	3
1	0	España	2	3	3	3	3
2	0	España	2	3	3	3	3
2	0	España	2	3	3	3	3
2	0	España	2	3	3	3	3
2	0	España	2	3	3	2	3
2	0	España	2	3	3	3	3
1	0	España	2	3	3	3	3
1	0	España	2	3	3	3	3

1	0	España	2	3	3	3	3
1	0	España	2	3	3	3	3
1	0	España	2	3	3	3	3
1	0	España	2	3	3	3	3
1	0	España	2	3	3	3	3
1	0	España	2	3	3	3	3
1	0	España	2	3	3	3	3
1	0	España	2	3	3	3	3
1	1	España	2	3	3	3	3
1	1	España	2	3	3	3	3
1	1	España	2	3	3	3	3
1	0	España	2	2	3	3	3
1	0	España	2	3	3	3	3
1	0	España	2	3	3	3	3
1	0	España	2	3	3	3	3
1	0	España	2	3	3	3	3
1	0	España	2	3	3	3	3
1	0	España	2	3	3	3	2
1	0	España	2	3	3	3	3
1	0	España	2	3	3	3	3
1	0	España	2	3	3	3	3
1	0	España	2	2	2	3	3
1	0	España	2	3	3	3	3
1	0	España	2	3	3	3	3
1	0	España	2	3	3	3	3
1	0	España	2	3	3	3	3
1	0	España	2	3	3	3	3
1	0	España	2	3	3	3	3
1	0	España	2	3	3	2	2
1	0	España	2	3	3	3	3
1	0	España	2	3	3	3	3
1	0	España	2	3	3	3	3
1	0	España	2	3	3	3	3
1	0	España	2	3	3	3	3
1	0	España	2	3	3	3	3
1	0	España	2	3	3	3	3
1	0	España	2	3	3	3	3
1	0	España	2	3	3	3	3
1	0	España	2	3	3	3	3
1	0	España	2	3	3	3	3
1	0	España	2	3	3	3	3
1	0	España	2	3	3	3	3
1	0	España	2	3	3	3	3
1	0	España	2	3	3	3	3
1	1	España	2	3	3	3	3
1	1	España	2	3	3	3	3
1	0	España	2	3	2	3	3
1	0	España	2	3	3	3	3
1	0	España	2	3	3	3	2

1	0	España	2	3	3	3	3
1	0	España	2	3	3	3	3
1	0	España	2	3	3	3	3
1	0	España	2	3	3	3	3
1	0	España	2	3	3	3	3
1	0	España	2	3	3	2	2
1	0	España	2	3	3	3	3
1	0	España	2	3	3	3	3
1	0	España	3	3	3	3	3
1	0	España	3	3	3	3	3
1	0	España	3	3	3	3	3
2	0	España	2	3	3	3	3
1	0	España	2	3	3	3	3
1	0	España	2	3	3	3	3
1	0	España	2	3	3	3	3
1	0	España	2	3	3	3	3
1	0	España	2	3	3	3	3
1	0	España	2	3	3	3	3
1	0	España	2	3	3	3	3
1	0	España	2	3	3	3	3
1	0	España	2	3	3	3	3
1	0	España	2	3	3	3	3
1	0	España	2	3	3	3	3
1	0	España	2	3	3	3	3
1	0	España	2	3	3	3	3
1	0	España	2	3	3	2	3
1	0	España	2	3	3	3	3
1	0	España	2	3	3	3	3
1	0	España	2	3	3	3	3
1	1	España	2	3	3	3	3
1	1	España	1	3	3	3	3
1	0	España	1	3	3	3	3
1	0	España	1	3	3	3	3
1	0	España	3	3	3	3	3
1	0	España	1	3	3	2	3
1	0	España	1	3	3	3	3
1	1	España	1	3	3	3	3
1	0	España	1	3	3	3	3
1	0	España	1	3	3	3	3
1	0	España	1	3	3	3	3
1	0	España	1	3	3	2	3
1	0	España	1	3	3	3	3
2	0	España	1	3	3	3	3
2	0	España	1	3	2	2	3
4	0	España	1	3	3	3	3
4	0	España	1	3	3	3	3
1	0	España	1	3	3	3	3
1	0	España	1	3	3	3	3
1	0	España	1	3	3	3	3
2	0	España	1	3	3	3	3

1	0	España	1	3	3	3	3
2	0	España	1	3	3	3	3
2	0	España	1	3	3	3	3
2	0	España	1	3	3	3	3
2	0	España	1	3	3	3	3
1	0	España	1	3	3	3	3
1	0	España	1	3	3	3	3
1	0	España	1	3	3	3	3
1	0	España	1	3	3	3	3
1	0	España	1	3	3	3	3
1	1	España	2	3	3	3	3
1	0	España	2	3	3	3	3
1	0	España	2	3	3	3	3
1	0	España	2	3	3	3	3
1	0	España	2	2	3	3	3
2	0	España	2	3	3	3	3
2	0	España	2	3	3	3	3
2	0	España	2	3	3	3	3
2	0	España	2	3	3	3	3
2	1	España	2	3	3	3	3
2	1	España	2	3	3	3	3
2	1	España	2	3	3	3	3
1	0	España	2	3	3	3	3
1	0	España	2	3	3	3	3
1	0	España	2	3	3	3	3
1	0	España	2	3	3	3	3
1	0	España	2	3	3	3	3
2	0	España	2	3	3	3	3
3	0	España	2	3	3	3	3
3	0	España	2	3	3	3	3
3	0	España	2	3	3	3	3
3	0	España	2	3	3	3	3
3	0	España	2	2	2	3	3
1	0	España	2	3	3	3	3
1	0	España	2	3	3	3	3
1	0	España	2	3	3	3	3
1	0	España	2	3	3	3	3
1	0	España	2	3	3	3	3
1	0	España	2	3	3	3	3
2	0	España	2	3	3	3	3
1	0	España	2	3	3	3	3
1	0	España	2	3	3	3	3
2	0	España	2	3	3	3	3
2	0	España	2	3	3	3	3
2	0	España	2	3	3	3	3
1	1	España	2	3	3	3	3
1	0	España	2	3	3	3	3
2	0	España	2	3	3	3	3
2	0	España	1	3	3	3	3
1	0	España	1	3	3	3	3

2	0	España	1	3	3	3	3
1	0	España	1	3	3	3	3
1	0	España	1	3	3	3	3
1	0	España	1	3	3	3	3
1	0	España	1	3	3	3	3
1	0	España	1	3	3	3	3
2	0	España	1	3	3	3	3
1	0	España	1	3	3	3	3
1	0	España	1	3	3	3	3
1	0	España	1	3	3	3	3
1	1	España	1	3	3	3	3
1	0	España	1	3	3	3	3
1	0	España	1	3	3	3	3
1	0	España	1	3	3	3	3
1	0	España	1	3	3	3	3
1	0	España	1	3	3	3	3
1	1	España	1	3	3	3	3
1	1	España	1	3	3	3	3
1	0	España	1	3	3	3	3
1	0	España	1	3	3	3	3
1	0	España	1	3	3	3	3
1	0	España	1	3	3	3	3
1	0	España	1	3	3	3	3
1	0	España	1	3	3	3	3
1	0	España	1	3	3	3	3
1	0	España	1	3	3	3	3
1	0	España	1	3	3	3	3
1	0	España	1	3	3	3	3
1	0	España	1	3	3	3	3
2	0	España	1	3	3	3	3
1	0	España	1	3	3	3	3
1	1	España	1	3	3	3	3
2	1	España	1	3	3	3	3
1	0	España	2	3	3	3	3
2	0	España	2	3	3	3	3
2	0	España	2	3	3	3	3
2	0	España	2	3	3	3	3
2	0	España	2	3	3	3	3
2	0	España	2	3	3	3	3
3	0	España	1	3	3	3	3
2	0	España	1	3	3	3	3
1	0	España	1	2	2	2	3
1	0	Estados Unidos	1	2	2	3	3
2	1	Estados Unidos	3	3	3	3	3
3	0	Estados Unidos	1	3	3	3	3
2	1	Estados Unidos	1	2	1	3	3
1	1	Estados Unidos	1	2	2	2	2
1	0	Estados Unidos	3	2	2	3	3
3	0	Estados Unidos	3	2	2	3	3
2	1	Estados Unidos	1	3	3	3	3

2	1	Estados Unidos	1	2	3	3	3
2	0	Estados Unidos	1	3	3	3	3
2	0	Estados Unidos	1	3	3	3	3
2	0	Estados Unidos	1	3	3	3	3
2	0	Estados Unidos	1	2	3	3	3
2	1	Estados Unidos	1	3	3	3	3
2	1	Estados Unidos	1	3	3	3	3
2	1	Estados Unidos	1	2	2	2	2
1	0	Estados Unidos	1	2	2	2	2
3	0	Estonia	3	3	3	3	3
1	0	Filipinas	1	3	3	3	3
1	1	Filipinas	1	3	3	1	3
1	1	Filipinas	1	1	3	1	3
1	1	Filipinas	1	1	3	3	3
2	0	Filipinas	1	1	3	3	1
3	1	Finlandia	1	3	3	3	3
3	1	Finlandia	1	3	3	3	3
2	1	Finlandia	1	3	3	3	3
3	1	Finlandia	1	3	3	3	3
3	0	Francia	1	3	3	1	3
2	0	Francia	1	2	3	1	3
2	1	Francia	1	2	3	3	2
2	1	Francia	1	3	3	3	3
3	0	Francia	1	3	3	2	3
3	1	Francia	3	3	3	3	3
2	0	Francia	2	3	3	3	3
1	0	Francia	1	3	3	3	1
3	0	Francia	1	3	3	3	3
3	0	Francia	3	3	3	2	3
2	0	Francia	3	2	3	3	3
3	1	Francia	1	3	3	3	2
3	0	Francia	1	3	1	3	3
2	0	Francia	1	2	2	3	3
3	1	Francia	1	3	3	3	1
2	1	Francia	1	2	2	3	3
2	0	Francia	1	3	3	3	3
3	0	Francia	1	3	3	3	3
2	0	Francia	1	3	3	3	2
3	0	Francia	1	2	3	3	3
3	1	Francia	1	3	3	3	3
2	1	Francia	1	3	3	3	3
2	0	Francia	1	3	3	3	3
3	0	Francia	2	3	3	2	2
3	1	Francia	2	3	3	3	3
2	0	Francia	2	3	3	2	2
3	0	Francia	1	3	3	3	3
3	0	Francia	1	3	2	2	3
3	0	Francia	1	3	3	3	3
2	0	Francia	1	3	3	3	3
2	0	Francia	3	2	2	2	2

2	0	Francia	3	3	3	3	3
2	0	Francia	2	2	3	3	3
2	0	Francia	3	3	3	3	3
2	0	Francia	3	3	3	3	3
3	0	Francia	3	3	3	3	3
1	0	Francia	2	3	3	3	3
1	0	Francia	2	3	3	3	3
1	1	Francia	2	2	2	3	3
1	1	Francia	2	3	2	3	3
1	0	Francia	2	3	3	2	3
1	0	Francia	2	3	3	3	3
1	0	Francia	2	3	3	3	3
1	0	Francia	1	3	3	3	3
1	0	Francia	1	3	3	3	3
1	0	Francia	2	3	3	3	3
1	0	Francia	2	3	3	3	3
1	1	Francia	2	3	3	3	3
2	1	Francia	1	3	3	3	3
3	0	Gabón	1	3	1	1	2
1	0	Gambia	1	3	3	2	2
3	1	Georgia	3	2	3	3	1
4	1	Georgia	3	3	3	3	2
3	1	Grecia	1	3	3	3	2
2	1	Grecia	1	3	3	3	2
3	1	Grecia	1	3	3	2	3
3	1	Grecia	1	2	3	3	3
3	1	Grecia	1	2	3	3	2
1	1	Grecia	2	3	3	3	3
2	1	Grecia	2	3	2	2	3
2	1	Grecia	2	2	3	3	3
3	1	Guatemala	1	3	3	3	2
3	1	Guatemala	1	2	3	3	3
2	1	Guatemala	1	2	3	3	3
3	1	Guinea Ecuatorial	1	3	3	3	3
3	1	Guinea Ecuatorial	1	3	3	3	3
3	1	Guinea Ecuatorial	1	1	3	3	3
3	0	Guinea Ecuatorial	1	3	1	3	3
1	0	Haití	1	1	3	1	3
2	1	Honduras	2	3	3	3	3
2	1	Honduras	2	2	2	2	2
3	0	India	1	3	3	1	3
3	0	India	1	3	3	3	3
3	0	India	1	3	3	1	1
3	0	India	1	3	3	3	1
3	0	India	1	3	3	2	3
3	1	India	2	3	3	3	2
2	0	Irlanda	2	3	3	3	3
3	1	Irlanda	3	3	3	3	3
2	1	Irlanda	1	3	3	3	1
2	1	Irlanda	3	3	3	3	3

3	0	Irlanda	3	3	3	3	1
3	1	Irlanda	3	3	3	3	3
2	1	Irlanda	1	3	3	3	3
3	1	Irlanda	1	3	3	3	3
3	1	Irlanda	1	2	2	2	3
2	0	Islandia	1	2	3	3	3
2	0	Islandia	3	2	3	3	3
3	1	Israel	3	3	3	3	3
1	0	Israel	1	2	3	3	3
3	0	Israel	1	2	2	3	3
3	1	Italia	1	3	3	3	3
3	0	Italia	1	3	3	3	3
3	0	Italia	3	2	3	3	3
3	0	Italia	3	2	3	3	3
3	0	Italia	1	3	3	3	3
1	0	Italia	1	3	3	2	3
3	0	Italia	1	2	2	3	3
2	1	Italia	3	2	3	3	3
1	0	Italia	1	2	3	3	3
1	0	Italia	1	2	3	3	3
1	0	Italia	3	2	3	3	3
2	0	Italia	1	2	3	3	3
3	1	Italia	2	3	3	3	3
2	0	Italia	1	3	3	3	3
3	0	Italia	1	3	3	3	3
3	1	Italia	1	3	3	3	3
3	1	Italia	1	3	2	2	2
2	0	Italia	1	2	2	2	2
2	0	Italia	1	3	2	2	2
2	1	Italia	3	3	3	3	2
2	1	Italia	2	3	3	3	2
2	1	Italia	1	3	3	2	2
3	0	Italia	1	2	2	3	3
2	0	Italia	1	3	3	3	2
1	0	Italia	1	3	3	3	3
1	0	Italia	1	3	3	3	3
1	0	Italia	1	3	3	3	3
2	0	Jamaica	1	3	3	2	2
3	1	Jamaica	1	3	2	2	2
3	0	Jamaica	1	3	2	2	2
3	1	Japón	1	3	1	3	3
3	0	Japón	1	3	3	1	3
4	0	Japón	1	3	3	3	3
3	0	Japón	1	3	2	1	1
2	0	Jordania	1	2	3	3	3
2	0	Jordania	1	2	3	2	3
3	0	Letonia	1	3	3	3	2
3	0	Líbano	1	3	3	3	2
3	0	Líbano	1	2	2	2	2
3	0	Líbano	1	2	2	3	3

2	1	Liberia	1	3	3	2	2
3	1	Liberia	1	1	3	3	2
1	0	Luxemburgo	3	3	3	3	3
3	0	Luxemburgo	3	3	3	3	3
2	0	Luxemburgo	1	3	3	3	2
2	0	Luxemburgo	1	3	3	3	3
3	0	Luxemburgo	1	3	2	3	1
2	1	Luxemburgo	1	2	3	3	3
3	0	Marruecos	3	3	3	3	2
2	1	Mauricio	1	3	3	3	3
2	0	Mauritania	1	3	3	2	2
4	0	México	1	3	3	2	2
2	1	México	1	2	2	2	3
2	1	México	1	2	2	2	2
2	1	México	3	2	2	3	3
2	1	México	1	3	3	3	3
3	1	México	1	2	2	2	2
2	0	México	1	3	2	2	2
3	0	México	1	3	3	3	3
2	0	México	1	3	3	3	3
3	0	México	1	3	3	3	3
2	0	Montenegro	3	3	2	3	3
2	1	Montenegro	1	2	2	1	1
3	1	Mozambique	1	3	2	2	2
4	0	Paraguay	1	2	2	3	3
2	0	Paraguay	1	2	3	3	2
2	1	Perú	1	2	2	3	3
1	1	Perú	1	3	3	3	1
3	1	Polonia	1	2	1	3	3
3	0	Portugal	1	3	3	3	2
3	0	Portugal	1	3	3	3	3
3	0	Portugal	2	3	3	3	2
3	1	Portugal	2	3	3	3	3
2	0	Portugal	1	1	1	3	3
3	0	Portugal	1	3	2	3	3
1	0	Portugal	1	2	3	3	3
2	1	Portugal	1	3	3	3	3
2	0	Portugal	1	2	3	3	3
2	0	Portugal	1	3	3	3	3
2	0	Portugal	1	2	2	2	3
2	0	Portugal	1	3	3	3	2
2	0	Portugal	3	3	3	3	3
2	0	Portugal	3	3	3	2	2
1	0	Portugal	1	3	3	3	3
1	0	Portugal	1	3	3	3	3
1	0	Portugal	1	3	3	3	3
1	0	Portugal	1	3	3	3	3
1	0	Portugal	1	3	3	3	2
1	0	Reino Unido	1	3	3	3	1
2	1	Reino Unido	1	3	3	3	3

2	1	Reino Unido	1	1	3	1	3
2	1	Reino Unido	2	3	3	2	2
3	0	Reino Unido	2	2	3	3	3
2	1	Reino Unido	3	2	3	3	3
1	1	Reino Unido	1	3	3	3	3
2	0	Reino Unido	1	3	3	3	3
2	0	Reino Unido	1	2	3	3	3
2	0	Reino Unido	2	2	3	3	3
3	0	Reino Unido	2	3	3	3	3
2	0	Reino Unido	2	2	3	3	3
2	0	Reino Unido	2	3	3	3	3
2	1	Reino Unido	2	3	3	2	2
3	1	Reino Unido	2	2	3	3	3
3	1	Reino Unido	2	3	3	3	3
2	1	Reino Unido	3	3	3	3	3
2	0	Reino Unido	3	3	3	3	3
1	0	Reino Unido	2	3	3	3	3
1	0	Reino Unido	2	2	3	3	3
1	0	Reino Unido	2	3	3	3	3
3	1	Rusia	1	3	3	3	3
2	1	Rusia	1	2	2	2	3
2	1	Sudáfrica	1	2	2	2	2
2	1	Sudáfrica	3	2	2	3	3
1	0	Sudáfrica	1	3	3	3	3
2	1	Suecia	1	2	2	2	3
2	0	Suecia	3	2	2	2	3
2	0	Suiza	1	3	3	3	3
2	0	Suiza	1	1	3	1	1
2	0	Suiza	3	3	3	3	3
3	0	Suiza	1	1	3	3	3
2	0	Suiza	1	2	3	3	3
2	0	Suiza	1	3	3	3	3
2	0	Suiza	1	3	3	3	3
2	1	Suiza	3	3	3	3	3
2	1	Suiza	3	3	3	3	3
1	0	Suiza	3	3	3	3	3
2	1	Suiza	3	3	3	3	3
3	1	Tailandia	1	1	2	3	3
3	0	Tailandia	1	1	3	3	3
1	0	Tailandia	1	3	3	1	1
1	0	Tanzania	1	3	1	1	2
4	1	Turquía	1	3	3	3	2
2	0	Turquía	1	3	3	3	2
2	0	Ucrania	1	1	3	3	3
3	1	Uganda	1	3	3	2	2
2	0	Uganda	1	3	2	2	2
2	0	Venezuela	1	3	2	2	2
2	0	Venezuela	1	3	2	2	2
3	0	Zimbabue	1	3	3	3	3
3	0	Zimbabue	1	3	3	3	2

4	1	Zimbabue	1	3	3	1	2

P05	P06	P07	P08	P09	P10	P11	P12
3	3	3	3	2	2	2	2
3	3	3	2	1	1	1	1
3	3	3	1	1	1	1	1
2	3	1	1	2	2	3	1
1	3	1	1	1	2	2	2
2	3	2	3	3	3	3	3
3	3	3	3	3	3	3	2
3	3	3	2	2	2	2	2
3	3	2	2	2	2	2	2
3	3	3	2	2	1	1	1
1	3	3	3	3	2	2	2
2	2	3	2	2	1	1	1
2	3	1	1	1	1	1	1
3	3	1	1	3	3	3	3
3	3	3	2	2	2	2	2
2	2	2	1	1	1	1	1
3	3	1	1	1	1	1	1
3	1	1	1	1	1	1	1
3	3	1	1	1	1	1	1
3	3	2	2	1	1	1	1
3	3	2	2	1	1	1	1
3	3	2	1	1	1	1	1
2	1	1	1	1	1	1	1
3	3	1	2	2	2	2	2
3	2	2	1	1	1	1	1
3	3	2	2	1	1	1	1
3	3	2	2	1	1	1	1
3	3	3	2	2	2	1	1
3	3	1	1	1	1	1	1
3	3	1	1	1	1	1	1
3	3	1	1	1	1	1	1
3	3	3	3	3	2	2	2
2	3	2	2	1	1	1	1
3	3	3	2	2	2	2	2
3	3	1	2	1	1	1	1
2	3	3	1	1	1	1	1
3	1	1	1	1	2	2	2
3	3	1	1	1	1	1	1
3	3	3	1	1	1	1	1
3	3	3	2	2	1	1	1
3	3	3	3	2	2	2	2
3	3	3	2	2	1	1	1
3	3	3	2	2	2	2	2
3	1	1	1	1	1	1	1
3	3	3	2	2	2	2	2
2	3	3	2	2	2	2	2
3	1	1	1	1	1	1	1
2	3	1	1	1	1	1	1
3	3	2	2	2	2	1	1

3	3	2	2	2	1	1	1
3	2	3	3	3	3	2	2
3	3	1	1	1	1	1	1
3	3	2	2	1	1	1	1
3	3	1	2	1	2	2	2
3	3	3	1	2	1	1	1
3	3	1	1	1	1	1	1
3	1	2	2	2	2	2	2
3	3	1	1	1	1	1	1
3	3	3	1	1	1	1	1
3	3	3	3	1	1	1	1
1	3	1	2	2	2	2	1
3	3	3	2	2	2	2	2
3	3	2	2	1	1	1	2
3	3	3	2	1	1	1	1
3	3	1	2	2	2	2	2
2	1	1	1	1	1	1	1
2	3	3	1	1	1	1	1
2	3	1	1	1	1	1	1
3	3	1	3	1	1	1	1
1	3	1	3	1	1	1	1
3	3	1	2	2	1	1	1
3	1	1	2	2	2	2	2
2	3	3	2	2	2	2	2
3	3	3	3	3	2	3	2
3	3	1	1	1	1	1	3
1	1	3	1	1	3	1	1
1	2	1	1	1	1	1	1
3	3	1	2	2	2	2	2
2	2	2	2	1	1	1	1
3	3	3	2	1	1	1	1
3	3	2	2	1	1	1	1
3	3	3	2	2	2	2	2
3	2	2	3	2	2	2	2
3	3	1	1	2	3	3	3
3	3	1	2	2	2	2	2
3	3	1	3	2	2	2	2
2	3	2	1	1	1	1	1
3	3	1	1	1	1	2	2
3	2	3	1	1	1	1	1
3	3	1	1	1	1	1	1
2	3	3	1	1	1	1	1
3	3	1	3	2	2	2	2
3	3	3	3	3	2	2	2
2	3	2	1	1	1	1	1
3	3	2	2	2	2	2	2
3	3	2	2	2	2	2	2
3	3	1	2	2	2	1	1
3	2	3	2	2	1	1	1
3	3	1	1	1	1	1	1

3	3	2	2	2	2	1	1
3	3	3	2	2	1	1	1
3	3	3	1	1	1	1	1
3	1	3	1	1	1	1	1
1	3	3	3	1	1	1	1
1	3	1	2	2	2	2	1
3	2	2	1	1	1	1	2
3	1	3	3	2	2	2	2
1	3	1	2	2	2	2	2
3	3	2	3	1	1	1	2
3	2	2	2	1	1	1	1
3	3	1	2	1	1	1	1
3	3	2	2	1	1	1	1
3	3	1	1	1	1	1	1
2	2	2	2	2	2	2	2
3	3	3	2	2	1	1	1
3	3	3	2	2	2	2	2
3	1	1	2	3	3	3	3
3	3	1	2	2	2	2	2
2	2	1	1	2	2	2	2
3	3	1	1	2	2	2	2
3	2	2	1	1	1	1	1
3	3	3	2	2	2	1	1
3	3	3	3	2	2	1	1
3	3	3	2	1	1	1	1
3	3	3	2	1	1	1	1
2	2	2	1	1	1	1	1
3	3	2	1	1	1	1	1
2	3	3	1	1	1	1	1
3	3	2	2	2	2	2	2
2	2	1	1	1	1	1	1
3	1	3	1	3	3	1	1
1	1	1	1	1	3	3	1
2	2	3	2	2	1	1	1
3	1	1	1	1	1	1	1
1	3	3	3	3	1	1	1
1	3	3	3	3	1	1	1
1	3	3	3	3	3	3	1
3	1	1	1	1	1	1	1
3	3	3	2	2	2	2	2
3	3	1	1	1	1	1	1
3	3	1	1	1	1	1	1
3	3	1	1	2	2	2	2
3	3	3	1	1	1	1	1
3	3	3	2	1	1	1	1
3	3	1	1	1	1	1	1
2	3	3	3	1	1	2	2
2	3	2	3	3	2	2	2
2	3	2	2	1	1	1	1
3	3	3	1	1	1	1	2

1	3	1	1	1	1	1	3
3	3	1	1	1	2	2	1
3	3	1	1	1	1	1	1
3	3	3	1	1	1	1	1
1	3	3	2	2	2	2	2
3	3	1	1	2	2	2	2
3	2	1	1	1	1	1	1
3	3	1	1	1	1	1	1
3	1	2	2	2	2	2	2
3	3	1	1	1	1	1	1
2	1	1	1	2	2	2	2
2	3	1	1	1	1	1	1
3	1	1	1	1	2	2	2
3	3	1	1	1	1	1	1
2	2	1	1	1	1	1	1
3	3	2	2	2	2	2	2
3	3	2	2	2	1	1	1
3	3	2	1	1	1	1	1
3	3	2	1	1	1	1	1
3	3	1	1	1	1	1	1
3	3	1	1	1	1	1	1
3	3	1	1	1	1	1	1
3	3	2	1	1	1	1	1
3	3	2	2	2	1	1	1
3	3	3	1	1	1	1	1
3	3	2	2	1	1	1	1
3	3	2	1	1	1	1	1
3	3	1	1	1	1	1	1
2	2	2	1	1	1	1	1
2	2	2	2	1	1	1	1
3	3	3	1	1	1	1	1
3	3	3	2	2	1	1	1
3	3	3	1	1	1	1	1
2	3	2	1	1	1	1	1
3	3	3	1	1	1	1	1
3	3	3	2	2	1	1	1
3	3	2	2	2	1	1	1
3	3	2	1	1	1	1	1
3	3	1	1	1	1	1	1
3	2	2	1	1	1	1	1
2	2	2	1	1	1	1	1
3	2	1	1	1	1	1	1
3	3	1	1	1	1	1	1
2	2	2	2	1	1	1	1
3	3	3	1	1	1	1	1
3	3	2	2	1	1	1	1
2	2	2	1	1	1	1	1
3	3	2	2	1	1	1	1
2	2	2	1	1	1	1	1
2	2	2	2	1	1	1	1

3	3	2	2	2	1	1	1
2	3	3	2	1	1	1	1
3	3	3	1	1	1	1	1
3	3	2	2	2	1	1	1
3	3	3	1	1	1	1	1
3	3	3	2	2	1	1	1
3	2	2	2	1	1	1	1
2	3	3	1	1	1	1	1
3	3	1	1	1	1	1	1
2	3	2	1	1	1	1	1
3	3	1	1	1	1	1	1
3	3	2	1	1	1	1	1
3	3	3	1	1	1	1	1
3	3	1	1	1	1	1	1
2	3	2	2	1	1	1	1
3	2	2	1	1	1	1	1
3	3	3	1	1	1	1	1
3	3	3	2	1	1	1	1
3	3	3	1	1	1	1	1
2	3	2	1	1	1	1	1
3	2	3	2	2	1	1	1
3	3	2	2	1	1	1	1
3	3	2	2	2	2	1	1
2	3	3	2	2	2	2	2
3	3	2	2	1	1	1	1
3	3	1	1	1	1	1	1
2	3	3	2	1	1	1	1
3	3	2	2	1	1	1	1
3	3	2	1	1	1	1	1
3	2	2	1	1	1	1	1
2	3	2	2	1	1	1	1
2	3	1	1	1	1	1	1
3	3	1	1	1	1	1	1
2	3	3	1	1	1	1	1
3	3	3	1	1	1	1	1
3	3	2	1	1	1	1	1
2	3	1	1	1	1	1	1
3	3	1	1	1	1	1	1
3	3	1	3	1	1	1	1
3	3	1	1	1	1	1	1
3	3	2	2	1	1	1	1
3	3	1	2	2	1	1	1
2	3	2	1	1	1	1	1
2	2	2	2	1	1	1	1
3	3	2	2	2	1	1	1
3	3	3	2	2	1	1	1
2	3	2	2	1	1	1	1
3	3	3	2	2	2	2	2
2	3	2	2	1	1	1	1
3	3	1	1	1	1	1	1

3	3	3	1	1	1	1	1
3	3	1	2	1	1	1	1
3	3	1	1	1	1	1	1
2	3	2	2	1	1	1	1
3	3	1	2	1	1	1	1
3	3	1	1	1	1	1	1
3	3	2	2	2	1	1	1
2	3	2	1	1	1	1	1
3	3	3	2	2	2	1	1
3	3	1	3	1	1	1	1
3	2	3	1	1	1	1	1
2	3	2	1	1	1	1	1
3	3	1	1	1	1	1	1
3	3	1	2	1	1	1	1
3	3	1	1	1	1	1	1
2	2	1	1	1	1	1	1
3	3	1	1	1	2	2	2
3	3	3	1	1	1	1	1
2	3	2	1	1	1	1	1
3	3	1	2	1	1	1	1
3	3	1	1	1	1	1	1
3	3	2	2	1	1	1	1
2	1	1	1	1	1	1	1
3	3	1	1	1	1	1	1
3	3	1	1	1	1	1	1
3	3	1	1	1	1	1	1
3	3	1	1	1	1	1	1
3	3	1	1	1	1	1	1
3	3	1	2	2	1	1	1
3	3	1	2	1	1	1	1
3	3	1	2	2	1	1	1
2	3	2	1	1	1	1	1
3	3	1	1	1	1	2	2
3	3	1	1	1	1	1	1
3	3	1	1	1	1	1	1
3	3	3	1	1	1	1	1
2	3	1	1	2	1	1	1
3	3	1	1	1	1	1	1
3	3	1	1	1	1	1	2
3	3	1	1	2	2	2	2
3	3	1	2	2	1	1	1
3	3	1	1	1	1	1	1
3	3	1	1	1	1	1	1
3	3	1	1	1	1	1	1
3	3	1	1	1	1	1	1
3	3	1	1	2	2	2	2
3	3	1	2	1	1	1	1
3	1	1	1	1	1	1	1
3	3	1	1	1	1	1	2
3	3	1	1	1	1	1	1

3	3	1	1	1	2	2	2
3	3	2	2	2	2	2	2
3	3	1	1	1	1	1	1
3	3	1	1	1	1	1	1
3	3	1	1	1	1	1	1
3	3	1	1	1	1	1	1
3	3	1	3	3	3	3	3
3	3	1	1	1	1	1	1
3	3	1	1	1	2	2	2
3	3	1	1	1	1	1	1
2	3	2	2	1	1	1	1
3	3	1	1	1	1	1	1
3	3	1	1	1	1	1	1
2	2	2	1	1	1	1	1
3	3	1	1	1	1	1	1
3	3	1	1	1	1	1	1
2	3	1	2	2	1	1	1
3	3	1	1	1	1	1	1
3	3	1	1	1	1	1	1
3	3	1	1	1	1	1	1
3	3	1	2	2	1	1	1
3	3	1	1	1	1	1	1
2	3	1	1	1	1	1	1
3	2	3	2	1	1	1	1
2	3	1	1	1	1	2	1
3	3	1	1	1	1	1	1
3	3	1	1	1	1	1	1
2	3	1	2	2	1	1	1
3	3	2	1	1	1	1	1
3	3	1	1	1	1	1	1
3	3	1	1	1	1	1	1
3	3	1	1	1	1	1	1
3	3	1	1	1	1	1	1
3	3	1	2	2	1	1	1
2	3	1	1	1	1	1	1
3	3	1	1	1	1	1	1
3	3	1	1	1	1	1	1
3	3	1	1	1	1	1	1
3	3	2	1	1	1	1	1
3	1	2	2	1	1	1	1
3	3	1	1	1	1	1	1
3	3	1	1	1	1	1	1
2	3	1	1	1	1	1	1
3	3	1	1	1	1	1	1
3	3	1	1	2	2	1	1
3	3	1	1	1	1	1	1
2	3	1	1	1	1	1	1
3	3	1	1	1	1	1	1
3	3	2	1	1	1	1	1
3	3	1	2	2	2	2	2

3	3	1	1	1	1	1	1
3	3	1	1	1	1	1	1
3	3	1	1	1	1	1	1
3	3	1	1	1	1	1	1
3	3	1	1	1	1	1	1
3	3	1	1	1	1	1	1
3	3	1	1	1	2	1	1
2	3	1	1	1	1	1	1
2	3	1	1	1	1	1	1
2	3	1	1	1	1	1	1
3	2	3	1	1	1	1	1
3	3	1	1	1	1	1	1
3	3	1	1	1	1	1	1
3	3	1	1	1	1	1	1
3	2	1	1	1	1	1	1
2	2	1	1	1	1	1	1
3	3	1	1	1	1	1	1
3	2	1	2	2	1	1	1
3	3	1	1	1	1	1	1
2	3	1	1	1	1	1	1
2	3	1	1	1	1	1	1
1	3	1	1	1	1	1	1
3	3	1	1	1	1	1	1
3	3	1	1	1	1	1	1
3	1	2	1	1	1	1	1
3	3	3	1	1	1	1	1
3	3	1	1	1	1	1	1
3	3	2	2	1	1	1	1
3	3	3	1	1	1	1	1
3	3	3	1	1	1	1	1
3	3	3	3	1	1	1	1
2	3	2	1	1	1	1	1
3	3	3	1	1	1	1	1
1	3	3	1	1	1	1	1
3	3	1	1	1	1	1	1
2	3	2	1	1	1	1	1
2	3	2	1	1	1	1	1
3	3	3	1	1	1	1	1
2	3	3	1	1	1	1	1
3	3	1	1	1	1	1	1
3	3	1	1	1	1	1	1
3	3	3	1	1	1	1	1
3	3	2	1	1	1	1	1
3	3	1	1	1	1	1	1
2	3	1	1	1	1	1	1
3	3	3	1	1	1	1	1
3	3	3	1	1	1	1	1
3	3	1	1	1	1	1	1
3	3	3	1	1	1	1	1
3	3	1	1	1	1	1	1

3	3	1	1	1	1	1	1
3	3	1	1	1	1	1	1
3	3	1	1	1	1	1	1
3	3	1	1	1	1	1	1
3	3	3	1	1	1	1	1
3	3	1	1	1	1	1	1
3	3	1	1	1	1	1	1
3	3	2	1	1	1	1	1
3	3	3	1	1	1	1	1
3	3	3	1	1	1	1	1
3	3	3	1	1	1	1	1
3	2	1	1	1	1	1	1
3	3	2	1	1	1	1	1
2	3	1	1	1	1	1	1
3	3	3	3	1	1	1	1
3	3	3	1	1	1	1	1
2	3	2	1	1	1	1	1
3	3	1	1	1	1	1	1
3	3	1	1	1	1	1	1
3	3	3	2	1	1	1	1
3	2	3	1	1	1	1	1
3	3	3	1	1	1	1	1
3	3	1	1	1	1	1	1
3	3	1	1	1	1	1	1
3	3	1	1	1	1	1	1
3	3	1	1	1	1	1	1
3	3	1	1	1	1	1	1
3	3	1	1	1	1	1	1
3	3	1	1	1	1	1	1
3	3	1	1	1	1	1	1
3	3	1	1	1	1	1	1
3	3	1	1	1	1	1	1
3	2	3	1	1	1	1	1
3	3	1	1	1	1	1	1
3	3	1	1	1	1	1	1
3	3	3	1	1	1	1	1
3	3	1	1	1	1	1	1
3	3	1	1	1	1	1	1
3	3	1	1	1	1	1	1
3	3	1	1	1	1	1	1
3	3	1	1	1	1	1	1
3	1	1	1	1	1	1	1
3	3	1	1	1	1	1	1
3	3	1	1	1	1	1	1
3	3	1	1	1	1	1	1
3	3	1	1	1	1	1	1
3	3	1	1	1	1	1	1
3	3	1	1	1	1	1	1
3	3	1	1	1	1	1	1
3	3	1	1	1	1	1	1

3	3	1	2	2	2	2	2
3	3	1	1	1	1	1	1
3	3	3	1	1	1	1	1
3	3	1	1	1	1	1	1
2	3	2	2	1	1	1	1
3	3	3	2	2	1	1	1
3	3	3	1	1	1	1	1
3	3	1	1	1	1	1	1
3	3	1	2	2	1	1	1
2	3	1	1	1	1	1	1
3	1	1	1	1	1	1	1
3	3	1	1	1	1	1	1
2	3	1	1	1	1	1	1
3	3	1	1	1	1	1	1
3	3	1	2	2	1	1	1
3	3	1	2	2	1	1	1
3	3	1	1	1	1	1	1
3	3	1	1	1	1	1	1
3	3	1	2	2	1	1	1
3	3	1	1	1	1	1	1
3	3	1	1	1	1	1	1
3	3	1	1	1	1	1	1
3	3	1	1	1	1	1	1
3	3	1	2	2	1	1	1
3	3	1	1	1	1	1	1
3	2	2	2	2	2	1	1
3	3	1	2	2	1	1	1
3	3	1	1	1	1	1	1
3	3	1	1	1	1	1	1
3	3	1	1	1	1	1	1
3	3	1	1	1	1	1	1
3	3	1	1	1	1	1	1
3	3	1	2	1	1	1	1
3	3	1	1	1	1	1	1
3	3	1	1	1	1	1	1
3	3	1	1	1	1	1	1
3	3	1	1	1	1	1	1
3	3	1	1	1	1	1	1
3	3	1	2	1	1	1	1
3	3	1	1	1	1	1	1
3	3	1	1	1	1	1	1
3	3	1	1	1	1	1	1
3	3	1	1	1	1	1	1
3	3	1	1	1	1	1	1
3	3	1	1	1	1	1	1
3	3	1	1	1	1	1	1
3	3	1	1	1	1	1	1
3	3	1	2	1	1	1	1
3	3	1	1	1	1	1	1
3	3	1	1	1	1	1	1

3	3	1	1	1	1	1	1
3	3	1	1	1	1	1	1
3	3	1	1	1	1	1	1
3	3	1	1	1	1	1	1
3	3	1	1	1	1	1	1
3	3	1	1	1	1	1	1
3	3	1	1	1	1	1	1
3	3	1	1	1	1	1	1
3	3	1	1	1	1	1	1
3	3	1	1	1	1	1	1
3	3	1	1	1	1	1	1
3	3	1	2	1	1	1	1
3	3	1	1	1	1	1	1
3	3	1	1	1	1	1	1
3	3	1	1	1	1	1	1
3	3	1	1	1	1	1	1
3	3	1	1	1	1	1	1
3	3	1	1	1	1	1	1
3	3	1	1	1	1	1	1
3	3	3	1	1	1	1	1
3	3	1	1	1	1	1	1
3	3	1	1	1	1	1	1
3	3	1	1	1	1	1	1
3	3	1	1	1	1	1	1
3	3	1	1	1	1	1	1
3	3	1	1	1	1	1	1
3	3	1	1	1	1	1	1
3	3	1	1	1	1	1	1
3	3	1	1	1	1	1	1
3	3	1	1	1	1	1	1
3	3	1	1	1	1	1	1
3	3	1	2	1	1	1	1
3	3	1	1	1	1	1	1
3	3	1	1	1	1	1	1
3	3	1	1	1	1	1	1
3	3	1	1	1	1	1	1
3	3	1	1	1	1	1	1
3	3	1	1	1	1	1	1
3	3	1	1	1	1	1	1
3	3	1	1	1	1	1	1
3	3	1	1	1	1	1	1
3	3	1	1	1	1	1	1
3	3	1	1	1	1	1	1
3	3	1	1	1	1	1	1
3	3	1	1	1	1	1	1
3	3	1	1	1	1	1	1
3	3	1	2	1	1	1	1
3	3	1	1	1	1	1	1
3	3	1	1	1	1	1	1
3	3	1	1	1	1	1	1

3	3	1	1	1	1	1	1
3	3	1	1	1	1	1	1
3	3	1	1	1	1	1	1
3	3	1	1	1	1	1	1
3	3	1	1	1	1	1	1
3	3	1	1	1	1	1	1
3	3	1	1	1	1	1	1
3	3	1	1	1	1	1	1
3	3	1	1	1	1	1	1
3	3	1	1	1	1	1	1
3	3	1	1	1	1	1	1
3	3	1	2	1	1	1	1
3	3	1	1	1	1	1	1
3	3	1	1	1	1	1	1
3	3	1	1	1	1	1	1
3	3	1	1	1	1	1	1
3	3	1	1	1	1	1	1
3	3	1	1	1	1	1	1
3	3	1	1	1	1	1	1
3	3	1	1	1	1	1	1
3	3	1	1	1	1	1	1
3	3	1	1	1	1	1	1
3	3	1	1	1	1	1	1
3	3	1	1	1	1	1	1
3	3	1	1	1	1	1	1
3	3	1	1	1	1	1	1
3	3	1	1	1	1	1	1
3	3	1	1	1	1	1	1
3	3	1	1	1	1	1	1
3	3	1	1	1	1	1	1
3	3	1	1	1	1	1	1
3	3	3	1	1	1	1	1
3	3	1	1	1	1	1	1
3	3	1	2	1	1	1	1
3	3	1	1	1	1	1	1
3	3	1	1	1	1	1	1
3	3	1	1	1	1	1	1
3	3	3	1	1	1	1	1
3	3	2	2	1	1	1	1
3	3	1	1	1	1	1	1
3	3	1	1	1	1	1	1
3	3	1	1	1	1	1	1
3	3	1	1	1	1	1	1
3	3	1	1	1	1	1	1
3	3	1	1	1	1	1	1
3	3	1	1	1	1	1	1
3	3	1	1	1	1	1	1
3	1	1	1	1	1	1	1
3	3	1	1	1	1	1	1
3	3	1	1	1	1	1	1

3	3	1	1	1	1	1	1
3	3	1	1	1	1	1	1
3	3	1	1	1	1	1	1
3	3	1	1	1	1	1	1
3	3	1	1	1	1	1	1
3	3	1	1	1	1	1	1
3	3	1	1	1	1	1	1
3	3	1	1	1	1	1	1
3	3	1	1	1	1	1	1
3	3	1	1	1	1	1	1
3	3	1	1	1	1	1	1
3	3	1	1	1	1	1	1
3	3	2	2	1	1	1	1
3	3	1	1	1	1	1	1
3	3	1	2	1	1	1	1
2	3	1	1	1	1	1	1
3	3	1	1	1	1	1	1
3	3	1	1	1	1	1	1
3	3	1	1	1	1	1	1
3	3	1	1	1	1	1	1
3	3	1	1	1	1	1	1
3	3	1	1	1	1	1	1
3	3	1	1	1	1	1	1
3	3	1	1	1	1	1	1
3	3	1	1	1	1	1	1
3	3	1	1	1	1	1	1
3	3	1	1	1	1	1	1
3	3	1	1	1	1	1	1
3	3	1	1	1	1	1	1
3	3	1	1	1	1	1	1
2	3	1	1	1	1	1	1
3	3	1	1	1	1	1	1
3	3	1	1	1	1	1	1
3	3	1	1	1	1	1	1
3	3	1	1	1	1	1	1
3	3	1	1	1	1	1	1
3	3	1	1	1	1	1	1
3	3	1	1	1	1	1	1
3	3	1	1	1	1	1	1
3	3	1	1	1	1	1	1
3	3	1	2	1	1	1	1
3	3	1	1	1	1	1	1
3	3	1	1	1	1	1	1
3	3	2	1	1	1	1	1
3	3	1	1	1	1	1	1
3	3	1	1	1	1	1	1
3	3	1	1	1	1	1	1
3	3	1	1	1	1	1	1
3	3	1	1	1	1	1	1
3	3	1	1	1	1	1	1

3	3	1	1	1	1	1	1
3	3	1	1	1	1	1	1
3	3	1	1	1	1	1	1
3	3	1	1	1	1	1	1
3	3	1	1	1	1	1	1
3	3	1	1	1	1	1	1
2	3	2	1	1	1	1	1
3	3	1	1	1	1	1	1
3	3	1	1	1	1	1	1
3	3	1	1	1	1	1	1
3	3	1	1	1	1	1	1
3	1	1	1	1	1	1	1
3	3	1	1	1	1	1	1
3	3	1	1	1	1	1	1
3	3	2	1	1	1	1	1
3	3	1	1	1	1	1	1
3	3	1	1	1	1	1	1
3	3	1	1	1	1	1	1
3	3	1	1	1	1	1	1
3	3	1	1	1	1	1	1
3	3	1	1	1	1	1	1
3	3	1	1	1	1	1	1
3	3	1	1	1	1	1	1
3	3	1	1	1	1	1	1
3	3	1	1	1	1	1	1
3	3	1	1	1	1	1	1
3	3	1	1	1	1	1	1
3	3	2	2	1	1	1	1
3	3	1	1	1	1	1	1
3	3	1	1	1	1	1	1
3	3	3	1	1	1	1	1
3	3	1	1	1	1	1	1
3	3	1	1	1	1	1	1
3	3	1	1	1	1	1	1
3	3	1	1	1	1	1	1
3	3	1	1	1	1	1	1
3	3	1	1	1	1	1	1
3	3	1	1	1	1	1	1
3	3	1	1	1	1	1	1
3	3	1	1	1	1	1	1
3	3	1	1	1	1	1	1
3	3	1	1	1	1	1	1
3	3	1	1	1	1	1	1
3	3	1	1	1	1	1	1
3	3	1	1	1	1	1	1
3	3	1	1	1	1	1	1
3	3	1	1	1	1	1	1
3	3	1	1	1	1	1	1
3	3	1	1	1	1	1	1
3	3	1	1	1	1	1	1

3	3	2	2	1	1	1	1
3	3	1	1	1	1	1	1
3	3	1	1	1	1	1	1
3	3	1	1	1	1	1	1
3	3	1	1	1	1	1	1
3	3	1	1	1	1	1	1
3	3	1	1	1	1	1	1
2	3	1	1	1	1	1	1
3	3	1	1	1	1	1	1
3	3	1	1	1	1	1	1
3	3	1	1	1	1	1	1
3	3	1	1	1	1	1	1
3	3	1	1	1	1	1	1
3	3	1	1	1	1	1	1
3	3	1	1	3	1	1	1
3	3	1	1	1	1	1	1
3	3	1	1	1	1	1	1
3	3	1	1	1	1	1	1
3	3	1	1	1	1	1	1
3	3	1	1	1	1	1	1
3	3	1	1	1	1	1	1
3	3	1	1	1	1	1	1
3	3	1	1	1	1	1	1
3	3	1	1	1	1	1	1
3	3	1	1	1	1	1	1
3	3	1	1	1	1	1	1
3	3	1	1	1	1	1	1
3	3	1	1	1	1	1	1
3	3	1	1	1	1	1	1
3	3	1	1	2	2	1	1
3	3	1	1	1	1	1	1
3	3	1	1	1	1	1	1
3	3	1	1	1	1	1	1
3	3	1	1	1	1	1	1
3	3	1	1	1	1	1	1
3	3	1	1	1	1	1	1
3	3	1	1	1	1	1	1
3	3	1	1	1	1	1	1
3	3	1	1	1	1	1	1
3	3	1	1	1	1	1	1
3	3	1	1	1	1	1	1
3	3	1	1	1	1	1	1
3	3	2	1	2	2	2	2
3	3	3	3	3	3	3	3
3	3	3	3	3	2	2	2
3	3	1	1	1	1	1	1
2	2	3	3	1	1	1	1
3	3	3	1	1	1	1	1
3	3	2	1	1	1	1	1
3	3	3	2	2	2	2	2

3	3	3	2	2	2	2	2
3	3	3	3	2	2	2	2
3	3	3	3	3	2	2	2
3	3	3	3	2	2	2	2
2	3	3	2	2	2	2	2
3	3	3	2	2	2	2	2
3	2	3	2	2	2	2	2
2	2	1	1	1	1	1	1
2	2	2	2	1	1	1	1
3	3	3	3	2	2	1	1
1	3	1	3	3	2	2	2
2	2	3	2	2	2	1	1
3	3	1	2	2	1	1	2
3	3	1	3	1	2	2	2
1	3	1	2	2	2	2	1
3	3	2	2	2	2	2	2
3	3	3	3	3	3	3	3
3	3	2	2	2	2	2	2
3	3	3	3	3	2	2	2
3	3	3	1	1	3	2	2
1	3	1	2	1	1	2	1
3	3	3	3	3	3	2	2
3	3	3	3	3	3	2	2
2	3	3	2	3	3	3	2
3	3	3	2	2	2	2	2
3	3	3	1	1	1	1	1
1	3	1	1	2	2	2	2
3	1	1	1	1	2	2	2
3	3	1	3	2	2	2	2
3	3	2	1	1	1	1	2
3	3	3	1	2	2	2	2
3	3	2	2	2	2	2	2
3	1	1	1	1	1	1	1
1	3	3	2	2	2	2	2
3	3	1	1	1	1	1	1
3	3	2	1	1	1	1	1
3	2	2	1	1	1	1	1
2	3	2	1	1	1	1	1
2	2	1	1	1	1	1	1
3	3	2	2	1	1	1	1
3	3	1	1	1	1	1	1
3	3	2	2	1	1	1	1
2	2	1	1	1	1	1	1
3	3	1	1	1	1	1	1
2	3	1	1	1	1	1	1
3	3	3	1	1	1	1	1
3	3	2	2	1	1	1	1
3	3	3	1	1	1	1	1
3	2	2	2	1	1	1	1
3	3	1	2	2	1	1	1

3	3	3	2	2	2	2	2
3	3	2	2	2	1	1	1
3	2	2	2	1	1	1	1
3	3	2	3	1	1	1	1
3	3	2	2	2	1	1	1
3	3	2	2	1	1	1	1
3	3	3	1	1	1	1	1
3	3	1	1	1	1	1	1
3	3	2	1	1	1	1	1
2	3	2	1	1	1	1	3
3	3	1	1	1	1	1	1
3	3	1	1	1	1	1	1
2	3	2	1	1	1	1	1
3	3	1	1	1	1	1	1
3	3	1	1	1	1	1	1
3	3	1	1	1	1	1	1
3	3	1	1	1	1	1	1
3	3	1	2	2	2	2	2
2	3	2	1	1	1	1	1
2	3	3	2	2	2	2	2
3	3	1	1	1	2	2	2
2	3	3	1	1	1	1	1
2	2	1	2	2	2	2	2
3	3	3	3	2	3	2	2
3	3	1	2	3	1	1	1
3	3	1	2	2	1	1	1
3	3	1	3	2	2	2	2
1	3	3	3	1	1	1	1
3	3	1	1	1	1	1	1
3	3	3	1	1	1	1	1
2	3	3	2	1	1	1	1
2	2	1	3	2	2	2	2
3	3	1	1	1	1	1	1
3	3	2	2	2	2	2	2
1	1	1	1	2	2	2	2
3	3	1	1	1	1	2	2
3	3	1	1	1	2	2	2
1	1	3	1	1	1	1	1
3	3	3	1	1	1	1	1
3	3	2	2	1	1	1	1
3	3	1	1	1	2	1	2
1	3	3	1	2	1	2	2
3	3	3	1	1	1	1	2
1	3	3	3	2	2	2	2
3	1	1	1	1	1	1	1
2	2	2	1	1	1	1	1
3	3	2	3	2	2	2	3
3	3	3	2	2	1	1	1
3	3	3	1	1	1	1	1
3	3	1	1	1	1	1	1

3	3	1	1	1	1	1	1
3	1	1	1	1	1	1	1
3	3	3	1	1	1	1	1
3	3	3	1	1	1	1	1
3	3	2	1	1	1	1	1
3	3	1	2	2	2	2	2
3	3	1	3	2	2	2	2
3	3	3	3	2	2	2	2
3	3	3	2	2	2	2	2
3	3	2	2	2	2	2	2
3	3	3	3	3	1	1	1
3	3	2	2	2	2	2	2
3	3	1	2	2	2	1	1
3	3	1	2	3	3	3	3
2	2	2	2	1	1	1	1
3	3	1	1	2	2	2	2
3	3	1	1	1	1	1	1
2	2	1	1	1	1	1	1
3	3	2	2	1	1	1	1
3	2	3	2	1	1	1	1
3	3	2	3	3	3	3	3
3	3	2	2	1	1	1	1
2	2	2	1	1	1	1	1
3	3	1	1	1	1	1	1
3	3	3	1	1	1	1	1
3	3	1	1	1	1	1	1
2	3	2	1	1	1	1	1
2	2	2	1	1	1	1	1
3	3	3	1	1	1	1	1
2	2	2	2	1	1	1	1
2	3	2	1	1	1	1	1
2	3	1	2	2	1	1	1
3	3	2	2	2	1	1	1
2	3	1	1	1	1	1	1
3	3	1	1	1	1	1	1
3	3	1	1	1	1	1	1
3	3	1	1	1	1	1	1
2	2	3	2	1	1	1	1
3	3	3	1	1	1	1	1
2	2	2	1	1	1	1	1
1	3	3	1	1	1	2	2
3	3	3	2	2	2	2	2
1	3	2	2	1	1	1	1
3	3	3	1	1	1	2	2
3	3	1	1	1	1	1	1
3	3	1	1	1	1	1	1
3	3	3	2	2	2	2	2
3	3	3	2	2	2	2	2
2	3	3	1	1	1	1	1
3	3	2	2	1	1	1	1

3	3	1	1	1	1	1	1
3	3	1	1	1	1	1	1
3	3	1	2	2	2	2	2
3	3	2	1	1	1	1	1
3	3	2	1	1	1	1	2
1	3	1	3	2	1	1	1
3	3	2	1	1	1	1	1
3	3	3	1	1	1	1	1
3	3	3	1	1	1	1	1
3	3	3	2	2	2	2	2
3	3	2	2	1	1	1	1
2	3	3	2	2	2	2	2
3	3	1	1	1	1	1	1
3	3	2	1	1	1	1	1
3	3	2	2	1	1	1	1
3	3	2	2	2	2	1	1
3	3	2	2	1	1	1	1
3	3	2	2	2	2	2	2
3	3	2	2	1	1	1	1
3	3	3	1	1	1	1	1
2	3	2	2	1	1	1	1
2	3	3	2	2	2	2	2
1	3	2	1	1	1	1	1
2	3	3	2	2	1	1	1
3	3	1	1	1	1	1	1
3	3	1	1	1	1	1	1
3	3	1	1	1	1	1	1
2	3	1	1	1	1	1	3
3	3	2	2	2	2	2	2
2	3	3	2	2	1	1	1
3	3	2	2	1	1	1	1
2	3	3	2	2	1	1	1
3	3	3	3	2	2	2	2
3	3	1	1	1	2	1	1
3	3	1	1	1	1	1	1
3	3	1	1	1	1	1	1
3	3	1	1	1	1	1	1
3	3	2	2	1	1	1	1
3	3	2	1	1	1	1	1
3	3	1	3	3	1	1	1
2	3	2	1	1	1	1	1
3	3	1	1	1	1	1	1
3	3	3	2	1	1	1	1
3	3	1	1	1	1	1	1
3	3	1	1	1	1	1	1
3	3	1	1	1	1	1	1
3	3	1	1	1	1	1	1
3	3	1	2	1	1	1	1
1	3	1	1	1	2	2	2
2	3	1	1	3	2	2	1

1	3	1	2	3	2	2	1
3	3	3	2	2	3	3	2
2	1	1	2	2	2	2	2
3	3	1	1	1	1	1	1
3	2	1	1	1	1	1	1
3	3	3	2	2	2	2	2
3	2	2	1	1	1	1	1
3	3	3	1	1	1	1	1
3	3	2	1	1	1	1	1
3	3	1	1	1	1	1	1
3	3	2	1	1	1	1	1
2	2	1	1	2	2	2	2
3	3	1	1	1	1	1	1
3	3	2	2	1	1	1	1
3	2	2	1	1	1	1	1
3	3	2	2	2	1	1	1
3	3	1	1	1	1	1	1
3	3	1	1	1	1	1	1
3	3	1	1	1	1	1	1
3	3	1	1	1	2	2	2
3	3	1	1	1	1	1	1
2	1	2	2	2	2	2	2
3	3	1	1	1	2	2	2
3	3	1	1	1	1	1	1
3	3	1	1	1	2	2	2
3	3	2	1	1	1	1	1
3	3	1	1	3	3	3	3
1	3	1	1	1	2	2	1
3	3	3	2	2	2	2	2
3	3	1	1	3	3	1	3
3	3	1	1	1	1	1	1
3	3	1	1	1	1	1	1
3	3	2	2	2	2	2	2
3	3	3	3	1	1	1	1
3	3	3	2	2	1	1	1
2	3	2	2	1	1	1	1
3	3	3	1	1	1	1	1
3	3	1	2	2	2	2	2
3	3	1	1	1	1	1	3
3	3	3	1	1	2	2	1
2	2	3	2	1	1	1	1
3	3	3	2	1	1	1	1
3	3	1	1	1	1	1	1
3	3	1	3	3	2	2	2
2	3	3	2	1	1	1	1
2	2	2	2	2	2	2	2
2	2	3	1	1	1	1	1
2	2	2	2	1	1	1	1
3	3	1	1	1	1	1	1
3	3	2	2	2	2	2	2

1	3	3	1	1	1	1	1
